# Supplementary figures and images for: Mating status drives fitness trade-offs in exercised female Drosophila
Source: PLoS One. 2025 Oct 24;20(10):e0327080. doi: 10.1371/journal.pone.0327080 (PMC12551838; doi:10.1371/journal.pone.0327080)

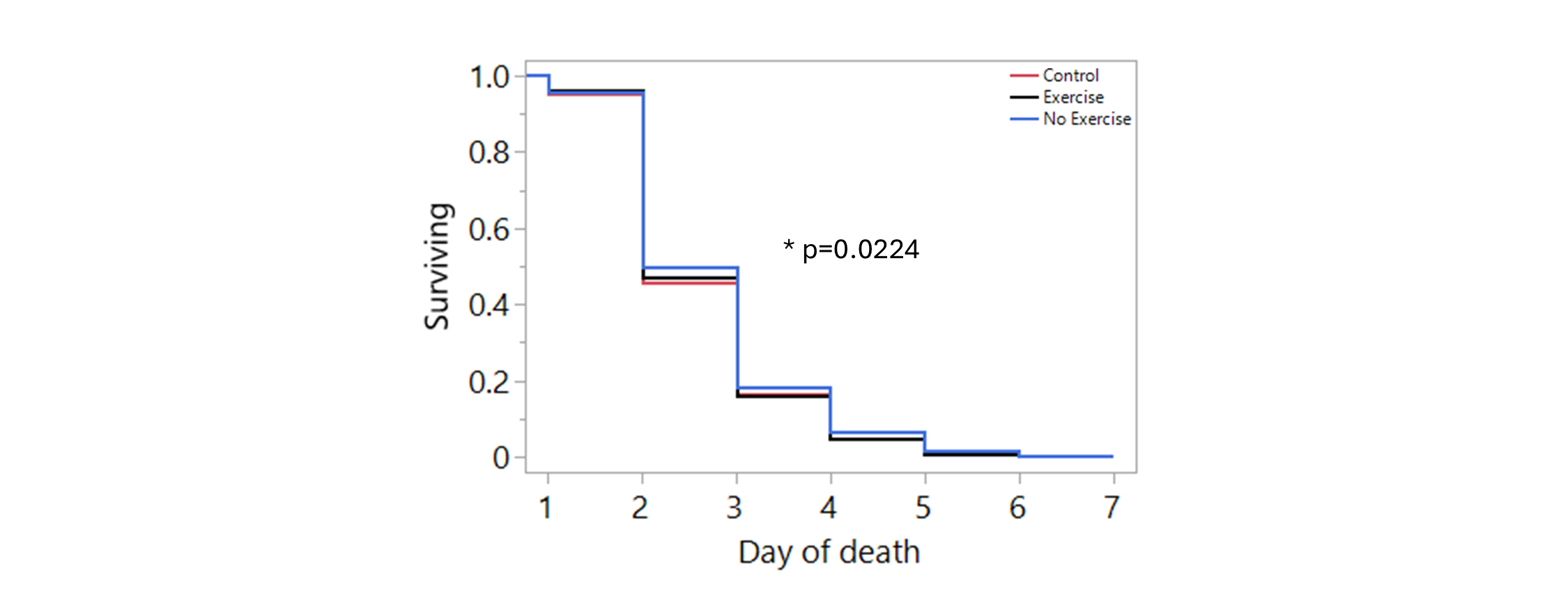

Supplement: S1 Fig — Starvation assay results for Exercised (red), Control (black), and No Exercise (blue) groups. The x-axis shows day of death since the beginning of the starvation assay (beginning at 13 days post-eclosion). (TIF) [file pone.0327080.s001.TIF]
